# Nonalcoholic fatty liver disease in women with polycystic ovary syndrome: associated factors and noninvasive fibrosis staging in a single Brazilian center

**DOI:** 10.20945/2359-3997000000242

**Published:** 2020-06-05

**Authors:** Daniela Oliveira de Lima Taranto, Thais Cristine Moura Guimarães, Claudia A. Couto, Ana Lúcia Cândido, Rosana Correa Silva Azevedo, Fernanda Souza Mattos, Maria Luiza Cândido Elias, Fernando M. Reis, Ana Luiza L. Rocha, Luciana C. Faria

**Affiliations:** 1 Serviço de Diagnóstico por Imagem Hospital das Clínicas Universidade Federal de Minas Gerais Belo Horizonte MG Brasil Serviço de Diagnóstico por Imagem do Hospital das Clínicas da Universidade Federal de Minas Gerais , Belo Horizonte , MG , Brasil; 2 Universidade Federal de Minas Gerais Belo Horizonte MG Brasil Pós-Graduação em Ciências Aplicadas à Saúde do Adulto, Universidade Federal de Minas Gerais , Belo Horizonte , MG , Brasil; 3 Instituto Alfa de Gastroenterologia Hospital das Clínicas Universidade Federal de Minas Gerais Belo Horizonte MG Brasil Instituto Alfa de Gastroenterologia , Hospital das Clínicas da Universidade Federal de Minas Gerais , Belo Horizonte , MG , Brasil; 4 Departamento de Clínica Médica Faculdade de Medicina Universidade Federal de Minas Gerais Belo Horizonte MG Brasil Departamento de Clínica Médica , Faculdade de Medicina da Universidade Federal de Minas Gerais , Belo Horizonte , MG , Brasil; 5 Serviço de Endocrinologia Hospital das Clínicas Universidade Federal de Minas Gerais Belo Horizonte MG Brasil Ambulatório de Hiperandrogenismo, Serviço de Endocrinologia , Hospital das Clínicas da Universidade Federal de Minas Gerais , Belo Horizonte , MG , Brasil; 6 Departamento de Ginecologia e Obstetrícia Faculdade de Medicina Universidade Federal de Minas Gerais Belo Horizonte MG Brasil Departamento de Ginecologia e Obstetrícia , Faculdade de Medicina da Universidade Federal de Minas Gerais , Belo Horizonte , MG , Brasil

**Keywords:** Polycystic ovary syndrome, nonalcoholic fatty liver disease, obesity, insulin resistance, liver fibrosis, infertility

## Abstract

**Objective:**

Polycystic ovary syndrome (PCOS) is a recognized risk factor for nonalcoholic fatty liver disease (NAFLD). The aims of this study were to investigate the prevalence and factors associated with NAFLD in women with PCOS and evaluate noninvasive indices of hepatic fibrosis in patients with PCOS and NAFLD.

**Subjects and methods:**

Patients with PCOS (n = 87) and women without PCOS (n = 40; controls) were included. NAFLD was diagnosed by abdominal ultrasonography after exclusion of alcohol consumption and viral or autoimmune liver disease. Anthropometric, clinical and metabolic variables, homeostasis model assessment of insulin resistance (HOMA-IR) index, lipid accumulation product (LAP), FIB-4 index, NAFLD score, and transient elastography (TE; FibroScan) were obtained in subsets of patients with PCOS and NAFLD.

**Results:**

A total of 87 patients with PCOS were included (mean age: 34.4 ± 5.7 years, mean body mass index [BMI]: 34.7 ± 4.7 kg/m ^2^ ). NAFLD was present in 67 (77.0%) patients with PCOS versus 21 of 40 (52.5%) controls (p = 0.005). Women with PCOS and liver steatosis, compared with their NAFLD-free counterparts, had higher values of BMI, waist circumference, triglycerides, total cholesterol, alanine and aspartate aminotransferases, and γ-glutamyltransferase, along with higher frequencies of obesity, metabolic syndrome, and insulin resistance. NAFLD was independently associated with waist circumference, serum triglycerides, and alanine aminotransferase levels. The FIB-4 index was not compatible with advanced fibrosis in any of the evaluated patients, while NAFLD score and TE were compatible with advanced liver fibrosis in 1 of 26 (3.8%) and 3 of 25 (12%) patients, respectively.

**Conclusion:**

Women with PCOS had a high risk of NAFLD, and a combination of both was associated with central obesity, dyslipidemia, insulin resistance, and metabolic syndrome. Noninvasive methods suggested low rates of severe hepatic fibrosis in Brazilian women with PCOS. Arch Endocrinol Metab. 2020;64(3):235-42

## INTRODUCTION

Nonalcoholic fatty liver disease (NAFLD) is a spectrum of clinical and pathological conditions that can manifest as simple steatosis, with accumulation of lipids in the liver parenchyma, or nonalcoholic steatohepatitis (NASH), characterized by hepatocyte injury, inflammation, and fibrosis in patients without significant alcohol consumption. NAFLD is the most common chronic liver disease in industrialized countries and is a leading cause of end-stage liver disease, hepatocellular carcinoma, and liver transplantation worldwide ( 1 , 2 ). It is also strongly associated with obesity, diabetes mellitus (DM), insulin resistance (IR), and metabolic syndrome (MS).

Polycystic ovary syndrome (PCOS) is one of the most common endocrine disorders in premenopausal women, affecting 6-20% of this population, depending on the diagnostic criteria adopted ( 3 ). The classic phenotype of the syndrome includes hyperandrogenism and ovulatory dysfunction, while the syndrome is strongly associated with obesity, IR, MS, and low-grade chronic inflammation ( 4 , 5 ). Multiple genetic, metabolic, and hormonal factors interact in PCOS.

There is increasing evidence of an association between NAFLD and PCOS ( 6 ). Obesity – mainly central adiposity – and IR are the main factors related to NAFLD in PCOS ( 7 ). Androgen excess, which is the main feature of PCOS and is related to IR, may be an additional contributing factor to the development of NAFLD in PCOS ( 8 , 9 ).

NASH and, most importantly, fibrosis severity have been strongly implicated in the long-term prognosis of NAFLD patients ( 10 ). During the last decade, noninvasive biomarkers have been developed and validated to rule out advanced fibrosis in patients, including the FIB-4 index ( 11 ) and NAFLD fibrosis score ( 12 ). However, little is known about the severity, comorbidities, and complications of NAFLD, such as liver fibrosis, in South American women with PCOS.

The current study was conducted to determine the prevalence, associated factors, and noninvasive fibrosis staging of NAFLD in a population of patients with PCOS at a single Brazilian center.

## SUBJECTS AND METHODS

### Subjects

We enrolled 87 consecutive nonpregnant women with a diagnosis of PCOS, who were attending the endocrinology clinic of the *Hospital das Clínicas* at *Universidade Federal de Minas Gerais* between September 2016 and November 2018. In addition, 40 nonpregnant women in reproductive age attending a gynecology clinic at the same hospital were recruited to serve as controls. PCOS was defined according to the Rotterdam criteria, which are based upon the presence of at least two of the following features: oligomenorrhea, hyperandrogenism, and polycystic ovaries ( 13 ).

The study was approved by the Ethics Committee of Human Research from *Universidade Federal de Minas Gerais* (090/2016). The subjects were excluded if they had a history of alcohol intake greater than 20 g per day or other chronic liver disease, or if using drugs that could cause liver steatosis.

### Clinical, anthropometric, and laboratory data

We prospectively obtained the participants’ medical history, including diagnosis of hypertension, DM, dyslipidemia, and a detailed history of alcohol consumption with an estimated daily intake in grams per day, followed by physical examination and blood tests. Anthropometric measurements included height, weight, body mass index (BMI), and waist circumference (WC). Obesity was defined as a BMI ≥ 30 kg/m ^2^ and central obesity as a WC ≥ 80 cm.

Clinical and laboratory data were recorded, including a modified Ferriman-Gallwey score ( 14 ) and phenotypic subgroup of PCOS, and levels of fasting plasma glucose (FPG), insulin, oral glucose tolerance test (OGTT), total testosterone (TT), total cholesterol (TC), high-density lipoprotein (HDL), low-density lipoprotein (LDL), triglycerides (TG), serum alanine aminotransferase (ALT), aspartate aminotransferase (AST), and γ-glutamyltransferase (GGT).

Patients with a diagnosis of PCOS were classified into four phenotypes according to the 2012 Methodology Workshop of the National Institute of Health ( 15 ): A (hyperandrogenism [HA] + ovulatory dysfunction [OD] + polycystic ovary [PCOM]), B (HA + OD), C (HA + PCOM), and D (OD + PCOM). The criteria proposed by the International Diabetes Federation (IDF) were used to define diabetes ( 16 ). Impaired fasting glucose (IFG) was defined as a FPG ≥ 100 mg/dL and < 126 mg/dL ( 17 ).

IR was estimated with the homeostasis model assessment of insulin resistance (HOMA-IR), calculated as fasting insulin (µUI/mL) x fasting glucose (mmol/L)/22.5. The lipid accumulation product (LAP) was calculated as (WC – 58) x TG (mmol/L) ( 19 ) and the visceral adiposity index (VAI) as:


$$\left(\frac{\mathrm{WC}}{36.58\;+\;\left(1.89\;x\;\mathrm{BMI}\right)\;\;}\right)\;\times \;\left(\frac{\mathrm{TG}}{0.81}\right)\;\times \;\left(\frac{1.52}{\mathrm{HDL}}\right)$$ (20)

The occurrence of IR was considered at a HOMA-IR > 2.7, ( 18 ) LAP ≥ 34.5, ( 19 ) and VAI > 1.675 ( 20 ).

The diagnosis of MS was defined according to the IDF criteria ( 16 ), which require the presence of WC ≥ 80 cm and two out of four factors: blood pressure ≥ 130/85 mmHg or the use of antihypertensive medication, FPG ≥ 100 mg/dL or a diagnosis of type 2 DM, HDL < 50 mg/dL or the use of lipid-lowering medication, and TG ≥ 150 mg/dL or the use of lipid-lowering medication.

Biochemical and clinical hyperandrogenism was previously defined at the moment of PCOS diagnosis using elevated TT levels and hirsutism assessed by the modified Ferriman-Gallwey score (patients with score ≥ 8 were considered to be hirsute) ( 14 ). Oligomenorrhea was defined as the occurrence of less than eight menstrual cycles per year and polycystic ovaries as more than 12 follicles per ovary or an ovarian volume > 10 mL on transvaginal ultrasonography ( 13 ).

Levels of ALT, AST, and GGT were deemed elevated when above the normal reference values provided by the laboratory (ALT > 69 IU/L, AST > 46 IU/L, and GGT > 43 IU/L).

### NAFLD diagnosis and staging

Abdominal ultrasonography was independently performed in all patients with PCOS by two radiologists with more than 15 years of experience in ultrasonography, who were unaware of the diagnosis of PCOS, using a 3.5 MHz convex probe (Toshiba Xario, Tochigi, Japan). Hepatic steatosis was considered absent when the echogenicity of the hepatic parenchyma was equal to that of the renal cortex. Fatty liver was determined in the presence of a higher echogenicity in the hepatic parenchyma compared with the renal cortex and with impaired visualization of the intrahepatic vessels and diaphragm. A diagnosis of liver steatosis was determined upon agreement of both examiners.

Women with PCOS and steatosis were assessed by a hepatologist and evaluated with clinical history taking, physical examination, and laboratory measurements, including liver enzymes, viral hepatitis serology, autoantibodies, protein electrophoresis, iron studies, ceruloplasmin, platelet count, alfa-1 antitrypsin, glycohemoglobin, and 25-hydroxyvitamin D. The NAFLD score and the FIB-4 index were calculated according to the following equations:


$$\mathrm{FIB}-4\;=\;\mathrm{Age}\;(\mathrm{years})\;\times \;\mathrm{AST}\;(U/L)\;/\;\mathrm{Platelets}\;\left(x10^{9}/1\right)\;\times \;\sqrt{\mathrm{ALT}}\;\left(U/L\right)$$ (11)

$$\mathrm{NAFLD}\;\mathrm{score}\;=\;-1.675\;+\;0.037\;\times \;\mathrm{age}\;\left(\mathrm{years}\right)\;+\;0.094\;\times \;\mathrm{BMI}\;\left(\mathrm{kg}/m^{2}\right)\;+\;1.13\;\times \;\mathrm{IFG}/\mathrm{diabetes}\;\left(\mathrm{yes}\;=\;1,\;\mathrm{no}\;=\;0\right)\;+\;0,99\;\times \;\mathrm{AST}/\mathrm{ALT}\;\mathrm{ratio}\;-\;0.013\;\times \;\mathrm{platelet}\;\left(\times 10^{9}/L\right)\;-\;0.66\;\times \;\mathrm{albumin}\;\;\left(g/\mathrm{dL}\right)$$ (12)

Transient elastography (TE), a noninvasive imaging method to evaluate hepatic fibrosis, was performed using FibroScan (Echosens, Paris, France) and an XL probe. Liver stiffness was expressed in kilopascal units (kPa), and the cutoff values of 7.0 kPa, 9.5 kPa, and 12.5 kPa were used to predict significant fibrosis, severe fibrosis, and cirrhosis, respectively (Metavir scores F2, F3, and F4, respectively) ( 21 ).

### Statistical analysis

Statistical analyses were performed using the Statistical Package for the Social Sciences (SPSS) for Windows, version 18.0 (SPSS Inc., Chicago, IL, USA). The Kolmogorov-Smirnov and Shapiro-Wilk tests were used to test the normality of the data. Continuous variables are presented as means and standard deviations (SDs) or medians and ranges, while dichotomous variables are presented as absolute numbers and percentages. Levene’s test was used to test for variance homogeneity.

Univariate logistic regression was performed to assess the association between steatosis and the following variables: age, BMI, WC, phenotype of PCOS, levels of TC, LDL, HDL, TG, TT, AST, ALT, GGT, HOMA-IR, LAP, VAI, and presence of hyperandrogenism, MS, IR, hypertension, DM, IGT, obesity, and central adiposity. Group differences for continuous variables were analyzed using Student’s t test or the Mann–Whitney U test and for categorical variables, using the chi-square test or Fisher’s exact test, as appropriate. Multivariate logistic regression was used to evaluate the association of independent factors with steatosis. Variables with a p value lower than 0.20 in the univariate analysis were included in the regression model as independent variables. Statistical significance was set at two-sided p values less than 0.05.

## RESULTS

In all, 91 patients with a diagnosis of PCOS were initially selected. Of these, we excluded one patient due to a prior diagnosis of hepatitis B and three who failed to show up for abdominal ultrasonography, totaling 87 patients. The control group included 40 participants.

### Prevalence of liver steatosis in the PCOS and control groups

The clinical, anthropometric, and laboratory characteristics of the participants in the PCOS and control groups are shown in Table 1 . The average age was lower in the PCOS group compared with the control group. There was no difference between the groups in relation to BMI, WC, prevalence of obesity, and prevalence of hypertension. The prevalence of DM was higher in the PCOS group. The prevalence of hepatic steatosis detected by ultrasonography was higher in the PCOS group (77.0%) than the control group (52.5%) (p = 0.005) ( Table 1 ).

**Table 1:** Clinical, anthropometric, and laboratory characteristics of the participants in the polycystic ovary syndrome and control groups

	PCOS (n = 87)	Controls (n = 40)	P value ^a^
Age (years)	34.4 ± 5.7	39.1 ± 7.6	0.001
BMI (kg/m ^2^ )	34.7 ± 4.7	33.8 ± 5.2	0.316
WC (cm)	103 (67-128)	104 (88-126)	0.310
Obesity – n (%)	75 (86.2)	31 (77.5)	0.220
Central obesity – n (%)	85 (97.7)	40 (100)	0.544
Hypertension – n (%)	22 (25.3)	11 (27.5)	0.400
DM – n (%)	11 (12.6)	3 (7.5)	0.019
Liver steatosis on ultrasonography – n (%)	67 (77.0)	21 (52.5)	0.005
Metabolic syndrome – n (%)	43 (49.4)	-	-
Dyslipidemia – n (%)	13 (14.9)	-	-
Hyperandrogenism – n (%)	66 (75.8)	-	-
IR (HOMA-IR ≥ 2.7) – n (%)	41 (47.1)	-	-
IR (LAP ≥ 34.5) – n (%)	72 (82.7)	-	-
IR (VAI ≥ 1.675) – n (%)	82 (94.2)	-	-
TG (mg/dL)	134 (49 - 373)	-	-
TC (mg/dL)	188 (127-288)	-	-
LDL (mg/dL)	116 (56-203)	-	-
HDL (mg/dL)	45 (23-77)	-	-
FPG (mg/dL)	90 (68-128)	-	-
ALT (U/L)	22 (11-81)	-	-
AST (U/L)	24 (9-48)	-	-
GGT (U/L)	36 (11-234)	-	-

PCOS: polycystic ovary syndrome; BMI: body mass index; WC: waist circumference; DM: diabetes mellitus; IR: insulin resistance; HOMA-IR: homeostasis model assessment of insulin resistance; LAP: lipid accumulation product; VAI: visceral adiposity index; TG: triglycerides; TC: total cholesterol; LDL: low-density lipoprotein; HDL: high-density lipoprotein; FPG: fasting plasma glucose; ALT: alanine aminotransferase; AST: aspartate aminotransferase; GGT: γ-glutamyltransferase.

### Clinical and anthropometric characteristics of patients in the PCOS group and factors associated with liver steatosis

The clinical, anthropometric, and laboratory characteristics of the participants with PCOS with and without associated NAFLD are shown in Table 2 . On univariate analysis, the presence of steatosis was associated with BMI/obesity, WC, levels of TG, TC, ALT, AST and GGT, OGTT result, and presence of IR (evaluated by LAP) and MS ( Table 2 ). On multivariate analysis, WC, serum triglycerides, and ALT levels were associated with the presence of hepatic steatosis ( Table 3 ).

**Table 2:** Clinical, anthropometric, and laboratory data of the participants with polycystic ovary syndrome with and without associated hepatic steatosis

	PCOS and hepatic steatosis (n = 67)	PCOS without hepatic steatosis (n = 20)	P value ^a^
Age (years)	35.0 ± 5.4	32.4 ± 6.4	0.07
BMI (kg/m ^2^ )	35.7 ± 4.1	31.2 ± 5.2	0.001
WC (cm)	103 (88-128)	95 (67-115)	< 0.001
Obesity n (%)	61 (91.0)	14 (70.0)	0.02
Central obesity	67 (100)	18 (90)	0.051
Hyperandrogenism – n (%)	53 (79.1)	13 (65.0)	0.13
Hypertension – n (%)	18 (26.9)	4 (20.0)	0.53
Metabolic syndrome – n (%)	39 (58.2)	4 (20.0)	0.007
IR (HOMA-IR ≥ 2.7) – n (%)	34 (50.7)	7 (35.0)	1.0
IR (LAP ≥ 34.5) – n (%)	61 (91.0)	11 (55.0)	0.001
IR (VAI ≥ 1.675) – n (%)	65 (97.0)	17 (85.0)	0.21
DM – n (%)	10 (14.9)	1 (5.0)	0.44
FPG (mg/dL)	89 (75-128)	85 (68-98)	0.06
OGTT (mg/dL)	123 (63-260)	107 (56-155)	0.005
Glycohemoglobin (mg/dL)	5.5 (4.9 - 6.9)	5.4 (4.8-5.7)	0.09
IFG (%)	12 (18.5)	0 (0)	0.06
Dyslipidemia n (%)	11(16.4)	2 (10.0)	0.72
TG (mg/dL)	140 (56-373)	92 (49-173)	0.003
TC (mg/dL)	195 (134 - 288)	177 (127-228)	0.01
LDL (mg/dL)	113 (59-203)	112 (56 -165)	0.17
HDL (mg/dL)	44 (23 - 77)	47 (33- 59)	0.48
Insulin (µIU/mL)	18 (8-55)	15 (3-37)	0.15
ALT (U/L)	36 (11-81)	25 (13-37)	0.001
AST (U/L)	24 (9-48)	18 (11-31)	0.001
GGT (U/L)	37 (14-234)	29 (11-68)	0.04
ALT ≥ 69 U/L n (%)	2 (2.9)	0 (0)	1.0
AST ≥ 460 U/L n (%)	2 (2.9)	0 (0)	1.0
GGT ≥ 44 U/L n (%)	23 (34.3)	2 (10.0)	0.32
Phenotype A	36 (53.7)	9 (45.0)	0.64
Phenotype B	6 (9.0)	3 (15.0)	
Phenotype C	11 (16.4)	2 (10.0)	
Phenotype D	14 (20.9)	6 (30.0)	

PCOS: polycystic ovary syndrome; BMI: body mass index; WC: waist circumference; IR: insulin resistance; HOMA-IR: homeostasis model assessment of insulin resistance; LAP: lipid accumulation product; VAI: visceral adiposity index; FPG: fasting plasma glucose; DM: diabetes mellitus; OGTT: oral glucose tolerance test; IFG: impaired fasting glucose; TG: triglycerides; TC: total cholesterol; LDL: low-density lipoprotein; HDL: high-density lipoprotein; ALT: alanine aminotransferase; AST: aspartate aminotransferase; GGT: gamma-glutamyltransferase; phenotypes: A (hyperandrogenism [HA] + ovulatory dysfunction [OD] + polycystic ovary [PCOM]), B (HA + OD), C (HA + PCOM), D (OD + PCOM).

**Table 3:** Results of multivariate logistic regression analysis of factors associated with hepatic steatosis in patients with polycystic ovary syndrome

Variable	OR	95% CI	P value ^a^
WC	1.013	1.002 – 1.024	0.025
TG	1.002	1.0 – 1.003	0.049
ALT	1.007	1.0 – 1.013	0.047

OR: odds ratio; 95% CI: 95% confidence interval; WC: waist circumference; TG: triglycerides; ALT: alanine aminotransferase.

Only two patients presented serum levels of aminotransferases above the upper limit of normality, and the levels of AST (univariate analysis) and ALT (univariate and multivariate analysis) were associated with an ultrasonographic diagnosis of hepatic steatosis.

### NAFLD staging

The FIB-4 index was calculated in 45 patients with PCOS and hepatic steatosis and had an average value of 0.56 ± 0.17. Considering all patients (100%), the FIB-4 index was lower than 1.3, which is considered a cutoff point to exclude advanced fibrosis, with a 90% negative predictive value ( 22 ).

**Figure 1:**
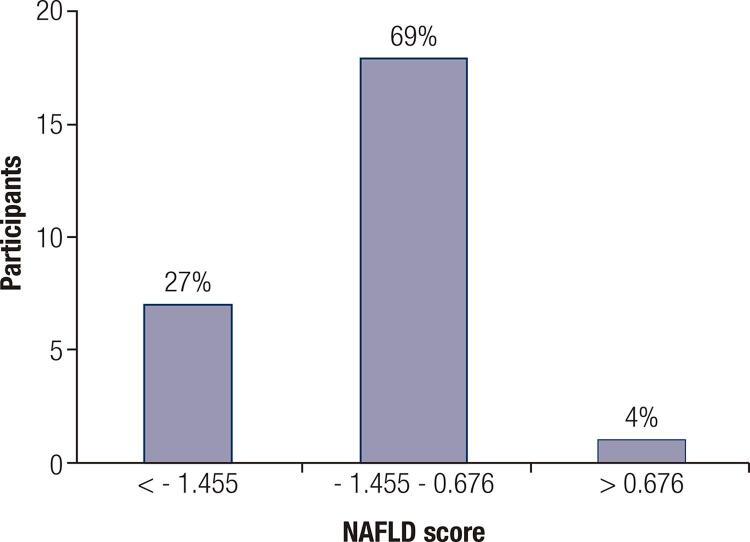
Distribution of the results of the nonalcoholic fatty liver disease (NALFD) score in participants in the polycystic ovary syndrome (PCOS) group with hepatic steatosis (n = 26).

The NAFLD score was calculated in 26 patients with PCOS and hepatic steatosis. In seven patients (27%), the score was lower than -1.455, which is the recommended cutoff value to rule out advanced fibrosis. Values considered indeterminate, between -1.455 and 0.676, were found in 18 patients (69%); in one patient (3.8%), the value was higher than 0.676, suggesting advanced hepatic fibrosis ( Figure 1 ).

**Figure 2:**
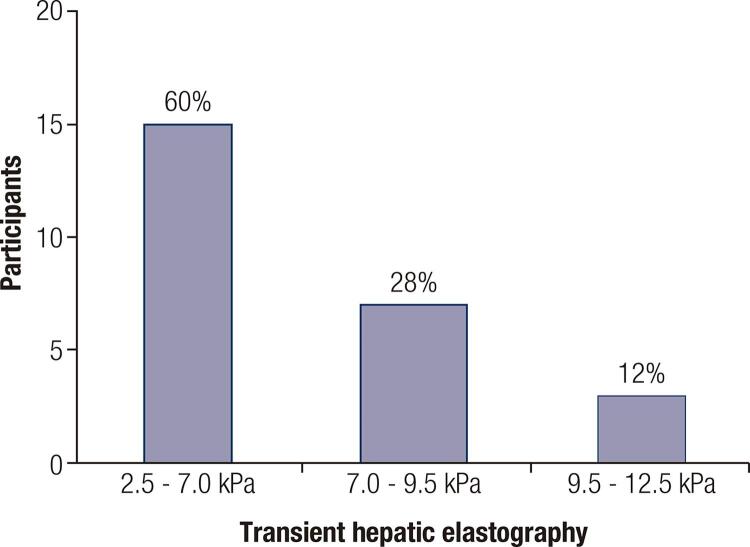
Distribution of the results of liver transient elastography in participants with polycystic ovary syndrome (PCOS) and hepatic steatosis (n = 25).

Liver TE was performed in 25 patients. Hepatic stiffness varied between 3.1 and 12.0 kPa, and was lower than 7.0 kPa (Metavir F0-F1, no significant fibrosis) in 15 patients (60%), between 7.0 and 9.5 kPa (F2, significant fibrosis) in seven patients (28%), and between 9.5 and 12.5 kPa (F3, severe fibrosis) in three patients (12%) ( Figure 2 ). One of the patients underwent liver biopsy and had no histological evidence of fibrosis (Metavir F0) or NASH (NAS score = 2, steatosis 1, ballooning 0, inflammation 1).

## DISCUSSION

In the present study, we compared the prevalence of hepatic steatosis in patients with PCOS *versus* a control group of women with similar BMI and WC and without a diagnosis of PCOS. Additionally, factors associated with the presence of steatosis in patients with diagnosed PCOS were investigated. We observed a high prevalence of liver steatosis in patients with PCOS (77.0%), which was associated with the following factors: WC, BMI/obesity, MS and IR diagnosed by LAP, and serum TG, TC, ALT, AST, and GGT. Multivariate analysis confirmed WC, TG, and ALT as independent factors associated with hepatic steatosis in patients with PCOS.

In another Brazilian study, the observed prevalence of NAFLD in patients with PCOS was 23.8% ( 23 ), a rate significantly lower than the one observed in the present study. This discrepancy may be due to different clinical and metabolic profiles of the studied populations. Compared with the characteristics of our population, the patients in the other Brazilian study were younger (26.8 ± 5 years), had a lower BMI (28.5 ± 6 kg/m ^2^ ), and a lower prevalence of MS (32.7%). Considering the impact of ethnic variability on the characteristics of PCOS and prevalence of NAFLD, as described by others ( 24 ), another possible explanation for the differences in age and BMI in both studies is the influence of ethnic-racial diversities in distinct Brazilian regions. Future studies may help to better elucidate the prevalence of NAFLD in patients with PCOS in different Brazilian populations.

The higher prevalence of hepatic steatosis observed in predominantly overweight patients with PCOS compared with a control group with similar BMI corroborates the hypothesis of the association between NAFLD and PCOS, which has also been described in eutrophic patients ( 25 ). On the other hand, comparing the two subgroups of PCOS with and without steatosis, patients with steatosis presented higher BMI, WC, and prevalence of obesity, which was consistent with previous studies ( 7 ). In a longitudinal study that evaluated the risk of NAFLD in 63,000 women with PCOS and in a control group including 121,000 women with similar age and BMI ( 26 ), women with PCOS presented an increased risk of NAFLD, which was higher than the expected due to obesity alone. Additionally, the diagnosis of PCOS was associated with a 2.0 to 2.4 times increased risk of NAFLD, including in nonobese women.

Although our sample was not population-based, the high prevalence of obesity in our patients with PCOS (86.2%) compared with that in most studies performed in other countries is a warning of the alarming growth of obesity in Brazil. The association observed between hepatic steatosis and central adiposity, triglyceride levels, and prevalence of MS corroborates previous reports ( 27 ). According to the knowledge that the components of MS are risk factors for NASH and development of advanced liver disease ( 28 ), the investigation of metabolic abnormalities in patients with PCOS is of great importance in clinical practice. The high prevalence and association of IR with steatosis confirm the importance of IR in the pathophysiology of both conditions. The frequency of diagnosis of IR was higher when LAP (82.7%) and VAI (94.2%) were used compared with HOMA-IR (47.1%). LAP was described in 2005 as a predictor of cardiovascular risk ( 29 ), and subsequent studies demonstrated its role in assessing the presence of IR in patients with PCOS ( 19 ). In addition to being used as a predictor of IR and visceral adiposity in patients with PCOS, VAI has been related to the risk of NAFLD in these patients ( 30 ). Interestingly, IR diagnosed by LAP was associated with NAFLD. IR is a common finding in women with PCOS, even in those with normal weight. Many mechanisms have been proposed for the development of IR in PCOS. The main one is a post-binding defect in insulin signaling due to increased serine phosphorylation and decreased tyrosine phosphorylation of the insulin receptor. IR in PCOs patients seems to be multifactorial and might reflect an influence of genetics, obesity, diet, and sedentary lifestyle. IR with hyperinsulinemia plays an important role in increased secretion of androgens and is an essential pathophysiologic mechanism in the development of all metabolic complications in these patients. Similar to PCOS, IR is intimately involved in the development of NALFD. IR results in increased hepatic *de novo* lipogenesis and impaired inhibition of adipose tissue lipolysis, with consequently increased flux of fatty acids to the liver. The cause and effect relationship between PCOS and NALFD is complex, and several studies have indicated IR as a fundamental link associating these conditions.

Most participants in our study had the PCOS phenotype A or B, which includes clinical or biochemical hyperandrogenism. As the present study was conducted in a reference center, the predominance of classic hyperandrogenic phenotypes may have been related to a selection bias, thus not representing the real distribution in the general population. Associations of steatosis with the classical phenotype and with hyperandrogenism, as described by other authors ( 9 , 31 ), were not detected in this study, possibly due to our limitation in adequately evaluating the occurrence of androgen excess. Hirsutism was present in 70.5% of the patients with PCOS, which is similar to previous studies ( 32 ). However, the quantification of hirsutism using the Ferriman-Gallwey score is affected by subjectivity, interobserver variability, and ethnic differences. Concerning the biochemical evaluation of hyperandrogenism, serum SHBG measurements were not available and were limited to total testosterone, which is less sensitive than the free androgen index to detect subtle hyperandrogenemia.

Only two patients (2.3%) with PCOS presented levels of aminotransferases above the upper limit of normal, both of whom had a diagnosis of NAFLD. Also, we observed an association between AST and ALT levels and the presence of hepatic steatosis. These findings demonstrate a low sensitivity of the routine cutoff values of serum aminotransferases in detecting NAFLD, despite the association between their increased levels and the diagnosis of NAFLD.

Although several studies have shown a high prevalence of steatosis in patients with PCOS, the detection of liver disease may still be underestimated. Despite having an acceptable level of sensitivity to detect liver fat, ultrasonography has some limitations, including a lower accuracy in the presence of obesity. Considering that 86.2% of the patients with PCOS in our study were obese, it is possible that the prevalence of liver steatosis in our cohort may have been even higher.

In NAFLD, fibrosis is the characteristic associated with the highest mortality risk; therefore, its early detection has great importance. In NAFLD staging in the present study, few patients with PCOS presented evidence of advanced liver fibrosis when evaluated with the FIB-4 index, NAFLD score, and TE. In contrast, these patients presented a high prevalence of risk factors for NASH and fibrosis progression, such as obesity and MS. The natural history of NAFLD in patients with PCOS is poorly understood. As in this study, other recent studies have used noninvasive methods like TE and serum biomarkers such as the FIB-4 index and NALFD score to estimate the liver disease stage more accurately in patients with PCOS ( 33 , 34 ).

Despite the evidence of a higher prevalence of NAFLD in women with PCOS and its potential for progressive liver disease due to the concomitance of MS factors, the association of NAFLD in patients with PCOS is not widely known by physicians taking care of these patients ( 35 ). In addition, the recommendations to investigate NAFLD in women with PCOS are controversial and the impact of NAFLD in these patients is most likely underestimated. A more precise definition of which factors are implicated in the pathophysiology of NAFLD in PCOS and knowledge of the natural history of liver disease in these patients may allow, in the future, a better selection of risk groups with more precise interventions.

The limitations of this study include the facts that alcohol consumption (considered as an exclusion criterion) was self-reported by the patients, the diagnosis of PCOS in the control group was not excluded by biochemical or imaging tests, and the results of laboratory tests were not available in all patients with PCOS. In addition, some patients with PCOS with an ultrasonographic diagnosis of steatosis did not comply with the proposed evaluation and staging of liver disease. Since this prevalence study recruited patients seeking medical evaluation due to symptoms related to PCOS, it is likely that the selected patients had more clinical features than the general population of patients with PCOS. Other limitations are inherent to a cross-sectional study, which fails to clarify whether the clinical conditions associated with the presence of NAFLD in concomitance with PCOS are etiological factors. Prospective cohort studies are required to establish the temporal sequence of events and elucidate the possible cause and effect relationship between PCOS and NAFLD.

In conclusion, the present study evaluating the association between NAFLD and PCOS in a Brazilian center demonstrated a high prevalence of steatosis in patients with PCOS when compared with patients with similar BMI and WC but without PCOS. Central adiposity and serum triglyceride levels, two components of the MS, were identified as independent factors associated with steatosis. Since MS is commonly related to progression of liver disease, patients with PCOS presenting central adiposity and increased triglyceride levels should be screened for NAFLD. Staging of liver disease using serum biomarkers and TE showed low stages of fibrosis in these patients, indicating that interventions in patients with premenopausal PCOS can be an important measure to reduce the risk of progression to advanced liver disease.
